# To Message or Browse? Exploring the Impact of Phone Use Patterns on Male Adolescents’ Consumption of Palatable Snacks

**DOI:** 10.3389/fpsyg.2017.02298

**Published:** 2018-01-08

**Authors:** Ethan Teo, Daniel Goh, Kamalakannan M. Vijayakumar, Jean C. J. Liu

**Affiliations:** ^1^Raffles Institution, Singapore, Singapore; ^2^Division of Social Sciences, Yale-NUS College, Singapore, Singapore; ^3^Neuroscience and Behavioural Disorders Programme, Duke-NUS Graduate Medical School, Singapore, Singapore

**Keywords:** technology, screen use, social facilitation, obesity, appetite

## Abstract

Surveys of mobile phone usage suggest that adolescents habitually use their phones while eating. In this study, we explored whether the manner in which one uses a mobile phone – to engage in a social or non-social activity – can affect appetite regulation. Participants were fifty male adolescents randomly assigned to engage in one of the following phone-based activities: (1) sending and receiving messages (social activity), or (2) reading a neutral article (non-social activity). When given the opportunity to snack, participants in the messaging group consumed more snacks that those who read the article. Our findings correspond to a large literature emphasizing social influences on food intake, and suggest that phone use patterns may predispose an individual to overeating.

## Introduction

Within the span of a decade, smartphones have permeated almost every aspect of our daily lives. Young adults report multi-tasking with their phones: in the restroom, during bedtime, waiting at a red light, and during meal-times (Webby Awards, 2015). Indeed, for one in four adolescents, phone use is a near-constant activity (Lenhart, 2015). Reflecting on this technological landscape, there have been recent efforts to develop guidelines for the use of mobile phones – particularly for the pediatric population growing up with ready access to smartphones (American Academy of Pediatrics, 2015; Radesky et al., 2015).

### Phone Use in an Obesogenic Environment

In the discussion of guidelines, one area of concern is the extent to which mobile phones may contribute to the obesogenic environment, predisposing children and adolescents to weight gain (Swinburn et al., 1999; AAP Council on Communications and Media, 2016a; Reid Chassiakos et al., 2016). Here, an analogy can be made to other forms of technology such as television and video games. For example, the increased consumption of television has been found to predict a higher body mass index and greater adiposity amongst children and adolescents (Coon and Tucker, 2002; Janz et al., 2002; Staiano et al., 2013). When given the opportunity to eat, those who do so while playing video games or watching television also show greater food intake (Temple et al., 2007; Chaput et al., 2011). Finally, interventions to decrease the use of television, videotapes, and video games have been successful in reducing the body mass index of school children (Robinson, 1999). Taken together, the current evidence suggests that using these technologies – collectively referred to as ‘screen time’ (for devices involving a screen) – constitutes a risk factor for obesity.

Although corresponding evidence for mobile phones is lacking, the American Academy of Pediatrics (2017) has classified phone usage as ‘screen time,’ generalizing findings from television and video games to mobile phones. This is reasonable in the discussion of weight management, since phone use – like other forms of screen use – is a sedentary activity (Lanningham-Foster et al., 2006). Additionally, multi-tasking with one’s phone has been found to be a distractor for tasks ranging from reading an article to crossing the road (Stavrinos et al., 2009; Chen and Yan, 2016). Since the primary account of why screen time promotes eating is that it distracts the user from satiety signals (Bellisle et al., 2004; Brunstrom and Mitchell, 2006; Hetherington et al., 2006; Robinson et al., 2013), multi-tasking with one’s phone can likewise be expected to increase food intake.

### Exploring the Social Nature of Phone Use

Beyond distraction, however, a key difference between smartphones and traditional forms of digital screens is that phone use is inherently social. Studies of phone use patterns consistently identify messaging functions as the top feature used in mobile phones (Lenhart, 2015; Smith, 2015), with adolescents estimating that they send 118 messages each day (Rideout et al., 2010). One implication of this usage pattern is that adolescents – when multi-tasking with their phones while eating – interact with friends and family in a way that they do not when multi-tasking with television or video games (with the exception of multi-player games).

The social nature of phone use is significant because individuals eat more with friends and family than they do alone – a phenomenon known as ‘social facilitation’ (de Castro and de Castro, 1989; de Castro, 1997; Herman, 2015). The mere company of one person can increase food intake by 44% (de Castro and de Castro, 1989; de Castro, 1997), with facilitation effects so robust that they have been observed: regardless of a person’s homeostatic hunger (de Castro and de Castro, 1989), regardless of the time and place of eating (de Castro et al., 1990), across groups of various cultures and demographics (Feunekes et al., 1995; de Castro et al., 1997), and across diverse study methodologies (Klesges et al., 1984; Berry et al., 1985; see de Castro, 1997; Herman et al., 2003; and Herman, 2015 for reviews of this literature).

Given the ubiquitous nature of social facilitation, a corollary question is whether phone-based messaging confers a risk for overeating – over and above the potential for phone use to distract the user. Although facilitation effects have traditionally been observed in the physical presence of other people, research on non-eating behaviors suggests that virtual presence may be sufficient (with social facilitation broadly defined here as the promotion of a dominant response; Zajonc, 1965). Thus, the virtual company of another person has been found to facilitate tasks ranging from anagrams, mazes, arithmetic, to exercise (Park and Catrambone, 2007; Anderson-Hanley et al., 2011; Snyder et al., 2012). Extending these findings, we investigated whether the virtual presence of friends and family – connected via phone-based messaging – would likewise result in the social facilitation of eating.

### The Current Study

To address this question, we conducted a randomized controlled trial monitoring the food intake of adolescents given the opportunity to snack. All participants used a mobile phone while eating, and differed only in how the phone was used: to engage in the social activity of sending and receiving messages (messaging group), or to carry out the non-social activity of reading a neutral article (control group). We hypothesized that messaging would result in the increased consumption of palatable snacks.

## Materials and Methods

### Participants

Participants were 50 male adolescents enrolled in Years 7–10 of an all-boys public school in Singapore (mean age: 14.64 years; *SD*: 0.75). We chose to recruit male participants as gender has been found to moderate phone use (Lenhart, 2015), eating behaviors (Wardle et al., 2004), and the relationship between technology and eating behaviors (Robinson and Killen, 1995); as such, including both genders would have required a much larger sample size. The study was conducted as part of the school’s research education program, and participants responded to school-wide advertisements inviting them to the study.

After written assent and written informed parental consent were obtained, participants were randomly allocated to either the messaging or control group. The two groups did not differ in age, ethnicity, body mass index, or baseline eating behavior (**Table 1**). All procedures were approved by the National University of Singapore’s Institutional Review Board (#A-15-170). All subjects gave written informed consent in accordance with the Declaration of Helsinki. The protocol was approved by the National University of Singapore.

**Table 1:** Baseline characteristics of participants allocated to the messaging and control groups.

	Experimental Group^1^		
		
Characteristic	Messaging (*n* = 25)	Control (*n* = 25)	Test statistic^2^ (*p*-value)
*Demographics*			
(a) Age (years)	14.68 (0.69)	14.60 (0.82)	-0.37 (0.71)
(b) Ethnicity	20 Chinese	22 Chinese	3.09^3^ (0.54)
	3 Indian	1 Indian
	1 Malay	1 Malay
	1 Others	1 Others
(c) Body mass index	21.53 (2.53)	21.05 (2.24)	-0.72 (0.48)
*Baseline eating behaviors*			
(a) Dutch Eating Behavior Questionnaire			
Restraint	2.47 (1.01)	2.30 (0.65)	-0.67 (0.50)
Emotional eating	2.28 (1.11)	2.24 (0.89)	-0.14 (0.89)
External eating	3.56 (0.75)	3.35 (0.67)	-1.04 (0.30)
(b) Time interval from previous meal (h)	4.34 (2.69)	4.84 (2.94)	0.63 (0.53)


*^1^Data reported as means (standard deviation) or counts. ^2^Unless otherwise stated, the test statistic refers to the *t*-statistic. ^3^Pearson’s chi-square statistic reported.*

### Materials

#### Baseline Questionnaires

As a measure of baseline eating behavior, we administered the Dutch Eating Behavior Questionnaire (DEBQ; van Strien et al., 1986). This questionnaire assessed whether participants ate based on: external rather than internal cues (‘external eating’), emotions (‘emotional eating’), or concerns to restrict one’s eating (‘restrained eating’). Reliability for each of the subscales was acceptable (Cronbach’s alpha for external eating = 0.77; emotional eating = 0.94; restrained eating = 0.88).

Additionally, we administered a questionnaire investigating participants’ use of social networking platforms. This asked participants which mobile phone they used, the number of friends they had on their phone contact list, the number of messages they sent each day, which social networking platforms they used, what they used their phone for, and whether they used their phone in everyday settings (in bed, in the toilet, during meals, in class, during commute, and during idle times).

#### Snack Food

For the snack food, we placed 50 g of chicken-flavored ‘Twisties’ (266 kcals; Mondelez International) in an unlabeled bowl. This corn puff snack was chosen because: (i) it is popular with adolescents, (ii) can be found in school vending machines, and (iii) comes in small regular-sized pieces. Pilot tests with a sample of students confirmed that the snack was palatable and that the portion size (50 g) exceeded what a typical student would consume in one setting.

### Procedure

Each experimental session took place at the end of a school day (mid-afternoon) and lasted for approximately 30 min. The set-up was intended to mimic what participants would typically encounter – the opportunity to eat highly palatable snacks following a day of school. On average, participants reported having eaten 4.5 h (*SD*: 2.8 h) before arrival (**Table 1**).

As the cover story, participants were made to believe that the researchers were interested in how technology influenced health. After completing baseline questionnaires, participants were told to bring out their mobile phones and to follow the experimenter’s instructions; additionally, they were told that they should not engage in any other activity with their phones. Compliance with phone use instructions was monitored through surreptitious observation from a distance.

In the messaging group, participants were asked to access the phone-based instant messaging service ‘WhatsApp.’ Within WhatsApp, participants identified an active chat group comprising of at least 10 users, and engaged in this group chat for a 10-min duration. Mimicking real-life situations, participants were given no other instructions regarding whom they should communicate with nor what they should discuss.

In the control group, participants were asked to access a neutral article sent to them via email. This was chosen to approximate web-browsing activities, implicated in phone use surveys as the top non-social function used on mobile phones (Rainie and Zickuhr, 2015). The article discussed a neutral topic (the immune system; MacPherson and Austyn, 2012), and was longer than what a typical student could finish reading during the session; additionally, 2 year 9 students who did not participate in the study assessed the article to be easy to read and neutral in tone. In short, this condition was comparable to previous distraction manipulations that had been found to increase food intake (e.g., listening to audio stories, listening to music, watching television; Bellisle and Dalix, 2001; Bellisle et al., 2004; Stroebele and de Castro, 2006; Long et al., 2011), and was designed to control for any distracting effects of mere phone use. Participants in this group read the article on their phones for a 10-min duration.

Across both conditions, the opportunity to eat was introduced in a casual manner. The bowl of snack food was left on the table throughout the 10 min, and the experimenter informed participants that the food was leftovers they were free to consume at will. At the end of the 10 min, participants were debriefed about the true aims of the study.

### Data Analyses

As the primary analysis, we ran an independent samples *t*-test comparing the amount of food consumed by participants in the messaging and control groups. The Type 1 error rate was controlled at α = 0.05, and power calculations showed that there was statistical power at the recommended 0.80 level to detect a large effect size (*d* = 0.80, comparable to effect sizes observed in previous social facilitation studies; Herman, 2015). All analyses were conducted using SPSS (IBM Corp., 2017) & R (R Core Team, 2017).

## Results

### Participants’ Baseline Patterns of Phone Usage

At baseline, 48% of participants reported regular use of their phones during meal-times (**Table 2**). Participants were most likely to use the messaging functions of their phones (**Table 3**), with 50% of participants sending at least 41 messages daily (**Table 4**). Together, these statistics suggest that the phenomenon being studied – texting while eating – is one participants themselves have likely engaged in on a regular basis.

**Table 2:** Messaging and control participants’ self-reported mobile phone usage during common activities.

	% Participants reporting phone usage during activity		
		
Activity	Messaging group (*n* = 25)	Control group (*n* = 25)	Chi-square (*p*-value)
Waiting or idle time (e.g., queuing in line)	92	80	1.50 (0.22)
During commute	68	56	0.76 (0.38)
Using the toilet	56	60	0.08 (0.77)
In bed	52	64	0.74 (0.39)
Eating a meal	48	48	0 (1.00)
Attending class	28	28	0 (1.00)


**Table 3:** Messaging and control participants’ self-reported use of mobile phone functions.

	% Participants reporting regular use of this function		
		
Phone function	Messaging group (*n* = 25)	Control group (*n* = 25)	Chi-square (*p*-value)
Sending messages	84	92	0.76 (0.38)
Browsing websites	80	80	0 (1.00)
Watching videos or listening to music	76	76	0 (1.00)
Playing games	64	80	1.59 (0.21)
Taking photos	68	72	0.10 (0.76)
Making phone calls	68	72	0.10 (0.76)


**Table 4:** Messaging and control participants’ frequency of sending mobile phone messages each day.

	% Participants reporting this frequency^1^	
	
No. of messages sent daily	Messaging group (*n* = 25)	Control group (*n* = 25)
≤10	20	16
11–20	20	4
21–30	8	16
31–40	12	4
41–50	8	8
>50	32	52


*^1^The distribution of participants did not differ significantly according to group; χ^*2*^(5, *N* = 46) = 6.51, *p* = 0.26.*

### Food Intake as a Function of Experimental Condition

#### Primary Analyses

**FIGURE 1:**
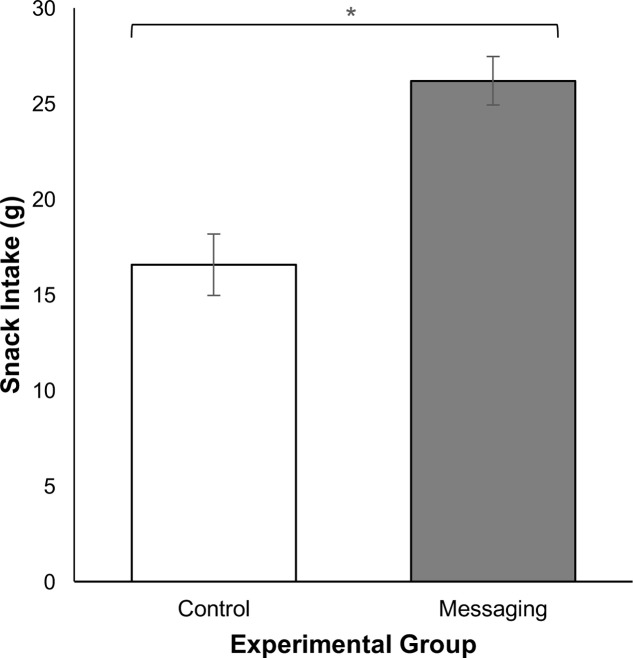
Snack intake of participants in the messaging and control groups; vertical lines represent 1 standard error of the means (^∗^*p* < 0.001).

As shown in **Figure 1**, participants in the messaging group consumed 58% more snacks than those in the control group [*t*(48) = -4.68, *p* < 0.001, *d* = 1.32]. The 95% confidence interval suggests that this corresponded to an average increase of 29.19–73.14 kcals consumed.

#### Accounting for Baseline Eating Behaviors

As a follow-up, we conducted a stepwise multiple regression to assess the influence of messaging after controlling for baseline eating behaviors. In Step 1, a model including: scores on the DEBQ (external, emotional, and restrained eating) and the time interval from the previous meal accounted for 5.2% of the variance in food intake, *F*(4,41) = 0.56, *p* = 0.69. Adding participants’ experimental condition in Step 2 explained a further 28.4% of the variance – a statistically significant increase [*F*(1,40) = 17.11, *p* < 0.001].

### Were Participants Primed or Distracted When Reading an Article?

Thus far, our results are consistent with the hypothesis that messaging activities would increase food intake relative to reading an article. However, an alternative account is the reverse – that reading the article reduced control participants’ snack consumption instead. This may have occurred if, instead of distracting participants, the topic of the article (the immune system) primed participants to eat in a healthy manner. This, in turn, may have caused them to eat fewer snacks.

Whereas distraction effects have been observed across various groups and situations, the influence of health primes is not universal (Forwood et al., 2015), affecting primarily dieters for whom primes reinforce their goals (Papies and Hamstra, 2010; Buckland et al., 2013; Papies, 2016). Correspondingly, if our article did indeed prime participants, those with higher dietary restraint would be more likely to show reduced food intake than those with low restraint. To this end, we ran a Pearson’s correlation between snack intake and DEBQ restraint scores amongst participants in the control condition. This correlation did not approach statistical significance [*r*(20) = 0.02, *p* = 0.92]. Similarly, amongst participants who had read the article, there was no significant difference in food intake between those with restraint scores at or above the median (≥2.3), as compared to those with scores below the median, [*t*(20) = 0.33, *p* = 0.74]. Taken together, we were unable to replicate a commonly observed pattern in the health priming literature, and found no evidence that priming mechanisms were at play.

## Discussion

In this study, we described the impact of smartphone messaging activities on appetite regulation. In line with research emphasizing social influences on food intake (de Castro, 1997; Herman, 2015), we found that male adolescents who sent and received text messages consumed more palatable snacks than those who used their phones to read an article. The difference between these two activities accounted for a third of the variance in snack consumption, and was a larger influence than time since the last meal or individual differences in eating behaviors (as measured by the DEBQ). To our knowledge, this is the first demonstration of how specific patterns of phone usage may predispose adolescents to over-eating.

### A Case for Virtual Social Facilitation

In terms of theory, our findings are consistent with ‘virtual social facilitation.’ Outside the field of ingestive behavior, several studies have found that social influence is so pervasive that computer-based or online presence is sufficient to elicit facilitation effects (Park and Catrambone, 2007; Anderson-Hanley et al., 2011; Snyder et al., 2012). Our study extends these findings to the eating domain, suggesting that the mere online presence of friends and family is able to promote eating behaviors.

At the same time, we caution that virtual social facilitation remains a nascent concept that requires follow-up. For example, the effects we observed do not fit neatly into current theories. By convention, social facilitation is classified based on what others are doing (Zajonc, 1965): ‘co-actors’ who are also eating cause the familiar increase in food intake, but a ‘passive (non-eating) audience’ renders the individual self-conscious – leading to a decrease in food consumption (Herman, 2015). With mobile phones, however, whomever one messages may not be a co-actor who is also eating. Similarly, message recipients are not privy to how much one eats, minimizing the need to maintain an impression via food intake. Accordingly, virtual company cannot be described to have either co-action or passive audience effects, and future research will need to investigate whether current accounts of social facilitation apply to the digital realm.

### Ruling Out Distraction Accounts

To strengthen the case for virtual social facilitation, future research will also need to rule out a solely cognitive explanation of our results. As described in the introduction, the primary account for why screen use affects food intake is that it diverts attention from the act of eating; with diminished cognitive resources, the screen-user engages in ‘mindless eating’ and consumes more (Ogden et al., 2013; Dohle et al., 2017). Although distraction effects were addressed through a control group engaged in a non-social phone activity, it remains possible that our activity – reading an article – was not as distracting to participants as messaging was. To the extent this was true, participants in the messaging group may have simply eaten more because they were more distracted (Bellisle et al., 2004; Brunstrom and Mitchell, 2006; Hetherington et al., 2006; Robinson et al., 2013) – rather than because the act of messaging was social in nature. Further studies are needed to tease apart these accounts by including other phone-use conditions (e.g., playing a solitary game), or by assessing cognitive resources required for messaging versus reading (e.g., through dual task paradigms).

### Toward Evidence-Based Guidelines on Pediatric Phone Use

More broadly, our findings add to the ongoing discussion of how technology contributes to the obesogenic environment. Beyond guidelines on whether or not digital screens should be used during meal-times (AAP Council on Communications and Media, 2016a,b), we found that the *manner* in which one uses a mobile phone can compound the problem of over-eating. This research is timely as our own participants reported the habitual use of mobile phones during a meal. While urging replication of our work, we tentatively suggest that switching from one of these activities (messaging) to the other (browsing and reading) could reduce the consumption of palatable snacks amongst adolescents.

### Study Limitations

Although we discuss the potential implications of our study, we highlight several limitations. First, our participants came from a homogenous all-boys school, and the extent to which these results generalize to other populations is unknown. Second, we chose to use an experimental design such that causality can be inferred. However, this required us to make several design choices that could limit generalizability. For example, we modeled our design on an everyday scenario where adolescents have the opportunity to snack after school. In so doing, we were focusing on the hedonic drive to eat, and are unclear whether similar results will be found when food intake is more strongly driven by homeostatic concerns (e.g., in a breakfast meal after an overnight fast; Lutter and Nestler, 2009). Similarly, in striving for ecological validity, we allowed participants in the messaging group to converse freely. This meant that we had little control over discussion topics, and cannot preclude the possibility that participants discussed the experiment in their chat groups (and perhaps were encouraged by their friends to eat). Finally, in the control condition, we opted to have participants use their phones for a non-social activity – reading an article. Although our analyses suggest that the article was unlikely to have attenuated food consumption (e.g., by priming a health message), we cannot rule out this possibility in the absence of a no-phone condition. In light of these limitations, we suggest that future research extend our findings through alternate operationalization of the experimental conditions. The use of diary or epidemiological designs would also allow the true impact of phone activities to be estimated amongst free-living adolescents.

## Conclusion

Our study was motivated by the observation that smartphones provide unprecedented opportunities for adolescents to connect with friends and family. Although the social feature of phones can have beneficial effects (Reid Chassiakos et al., 2016), choosing to message while eating can promote the overconsumption of food. Over time, this may predispose adolescents to weight gain, and is a potential risk factor that requires further study.

## Author Contributions

ET and DG conceptualized and designed the study, collected the data, drafted the initial manuscript, and approved the final manuscript as submitted. KV and JL conceptualized and designed the study, carried out statistical analyses, reviewed and revised the manuscript, and approved the final manuscript as submitted.

## Conflict of Interest Statement

The authors declare that the research was conducted in the absence of any commercial or financial relationships that could be construed as a potential conflict of interest.
